# Effect of Ultraviolet C light disinfection on the dimensional stability of dental impression materials: A scoping review of the literature

**DOI:** 10.4317/jced.62119

**Published:** 2024-11-01

**Authors:** Violeta Malpartida-Carrillo, Pedro Luis Tinedo-López, Julio Enrique Salas-Quispe, María Angélica Fry-Oropeza, Silvia Amaya-Pajares, Mutlu Özcan

**Affiliations:** 1 DDS, MSc. Professor, School of Stomatology, Universidad Privada San Juan Bautista, Lima, Perú; 2 DDS, MSc. Associate Professor of the Division of Oral Implantology, Department of Postgraduate in Stomatology, Universidad Científica del Sur, Lima, Perú; 3 DDS, MSc. Associate Professor, Department of Restorative Dentistry, School of Dentistry, Oregon Health and Science University, Portland, USA; 4 DDS, PhD. Professor, Center for Dental Medicine, Clinic for Masticatory Disorders and Dental Biomaterials, University of Zurich, Zurich, Switzerland

## Abstract

**Background:**

Ultraviolet C (UVC) light is a physical method proposed for disinfecting dental impression materials and preventing cross-infections in clinical practice. The investigations have focused on the UVC disinfection potential, but little is known about the consequences on dental materials’ properties. This scoping review’s objective is to evaluate information about the effect of UVC light on the dimensional stability of dental impression materials.

**Material and Methods:**

An electronic search of dental literature in the Medline (via PubMed), Scopus, and Embase databases were systematically searched until July 31 of 2024 following the PRISMA-ScR guidelines. The search strategy was carried out considering three groups of words with indexing terms and Boolean operators. Two reviewers selected the titles and analyzed the abstracts according to the inclusion and exclusion criteria.

**Results:**

A total of six articles were included through electronic database searches. Four studies evaluated the dimensional stability by measuring dental casts made from an impression and two studies measured distances on discs made from stainless steel dies. The studies reported the use of polyether, addition and condensation silicones, alginate, and zinc oxide eugenol in the protocols followed. Three of the six included studies compared the effect of UVC light against glutaraldehyde 2% and sodium hypochlorite (1% or 5.25%), one study compared the UVC light against quaternary ammonium salts, phenoxyethanol, alcohol, and ozone, meanwhile another study compared the effect of UVC light against peracetic acid 0.2%, natural polymer of glucosamine and ozonated water. Regarding measuring devices, only one study reported the use of a measuring software, the majority used traveling microscope.

**Conclusions:**

Based on the findings, UVC light showed no significant dimensional changes in polyether, addition, and condensation silicones.

** Key words:**Disinfection, dental impression materials, ultraviolet light.

## Introduction

Dental impressions are negative imprints of oral tissues (teeth, gingival tissues, and alveolus) used for making accurate casts of the dentition and its neighboring tissues capable of recording the prepared tooth and the surrounding anatomic topography of the desired area (1,2). Because dental impressions allow the creation of mouth structures replicas and patients’ teeth, they play a crucial role in the accurate and adequate diagnosis, fabrication of definitive restorations, and precise manufacturing of different types of oral appliances used in the dental specialties (3-5).

During dental impression procedures, dental impression materials come into contact with blood, debris, dental plaque, and saliva, which contain potentially pathogenic microorganisms and viruses (6). In the dentistry, there is evidence regarding the pathogenesis and intensity of tuberculosis, herpes simplex, hepatitis B and C, and AIDS viruses (7-9). Thus, without adequate disinfection, contaminated dental impressions can be a source of cross-infection between patients, dentists, dental assistants, and laboratory technicians (2). Hence, every impression should be first rinsed with water to remove all particles, blood, dental plaque, and saliva followed by disinfection before pouring casts because microorganisms can survive on or even inside the impressions (10).

Classically, based on the properties after the material has set, the dental impression materials can be classified as elastic and inelastic. Impression waxes, impression compound, impression plaster, and metallic oxide pastes are considered as inelastic impression materials; meanwhile, reversible hydrocolloid, irreversible hydrocolloid, polysulfide, polyether, condensation silicone, addition silicone (polyvinyl siloxane), and vinyl polyether siloxane are considered as elastic impression materials (4). The dental impression materials need to be adequately disinfected, and the effort to eliminate as many potential risks as possible is logical. It is essential to point out that, in view of the disinfectants, the investigations are oriented towards two main areas, considered as the main requirements for a disinfectant: a) the efficiency of the disinfecting solutions in eliminating the pathogens and b) the influence of the disinfection treatment on the impression material properties (11). In this regard, the properties that could be affected by the disinfection procedures are dimensional alterations commonly termed stability and accuracy, and the surface properties such as wettability, surface roughness and detail reproduction (12).

Dimensional stability refers to maintaining the size and shape of a material and is linked to dimensional changes related to setting or hardening (13). The dimensional changes of the impression materials may affect the fit quality and retention of the dental prostheses, which influence the success of the dental treatments. According to the literature, an ideal impression material should be dimensionally sTable, hydrophilic, flexible, reproducible, have good elastic recovery, have better flow properties and mechanical strength, and retain the imitation accuracy of intraoral imprints (14). Furthermore, the ideal impression material should meet appropriate setting time, be easily manipulated, be compatible with cast materials, low-cost, safe, and have desinfectability. From a practical standpoint, no material meets all the characteristics described above, but this is the current information available.

Several methods have been proposed for dental materials disinfection, such as disinfection by immersion or spraying technique, ethylene oxide, autoclave, microwave, ozone, electrolyzed oxidizing water, and ultraviolet (UV) radiation (10,15). Ultraviolet radiation uses the UV light that is divided in four types: UVA (wavelength: 315-400 nm), UVB (wavelength: 280-315 nm), UVC (wavelength: 200-280 nm), and vacuum UV (wavelength: 100-200 nm) (16). Ultraviolet C (UVC) light has been shown to drastically reduce microbes, including bacteria, fungi, yeasts, and viruses, and is considered a method to disinfect dental impressions because its performance has been demonstrated in investigations available in the literature (17,18).

In such a context, UVC light has been used in dentistry for disinfect impression materials (17,18), dental environments (19), contaminated toothbrushes (20), acrylic resins (21), and dental implants (22). Considering the dental impression materials, most investigations focused on disinfection, and little is known about the effect of the UVC light on the dental materials’ properties.

Hence, the objective of this scoping review is to evaluate comprehensively the information about the effect of UVC light on the dimensional stability of dental impression materials.

## Material and Methods

The Medline (via PubMed), Scopus, and Embase databases were systematically searched until July 31st 2024 considering the Preferred Reporting Items for Systematic Review and Meta-Analysis Extension for Scoping Reviews (PRISMA-ScR) guidelines (23). The search strategy was carried out considering indexing terms and the following group of words: Dimensional stability, dental impression materials, and ultraviolet light, that were combined with the Boolean operators “OR” and “AND”. Thus, the final strategy considered was: ((((dimensional stability) OR (dimensional accuracy)) OR (dimensional change)) AND ((((((((((((((((impression materials) OR (dental impressions)) OR (dental impression materials)) OR (impression wax)) OR (impression compound)) OR (impression plaster)) OR (metallic oxide paste)) OR (reversible hydrocolloid)) OR (irreversible hydrocolloid)) OR (alginate)) OR (polysulfide)) OR (polyether)) OR (condensation silicone)) OR (addition silicone)) OR (polyvinyl siloxane)) OR (vinyl polyether siloxane))) AND (((((((((ultraviolet light radiation) OR (ultraviolet light)) OR (ultraviolet radiation)) OR (UV radiation)) OR (UVC radiation)) OR (UV rays)) OR (UVC rays)) OR (UV chamber)) OR (UVC chamber)). In addition, reference lists of all included articles were also reviewed.

The inclusion criteria for selecting articles included were laboratory (*in vitro*) studies, studies based on dental impression materials, studies that reported results before and after disinfection based on UVC light, studies that showed the UV light wavelength used or the name/reference of the producing company to check the wavelength, and studies published in any language. The studies that satisfied one of the following criteria were excluded such as human, animal, review, or finite element studies, studies that did not inform their measurement results, and studies that reported only antimicrobial activity. Two reviewers (VMC and PLTL) assessed the included studies independently, and any disagreement was resolved by consensus.

## Results

**Figure 1:**
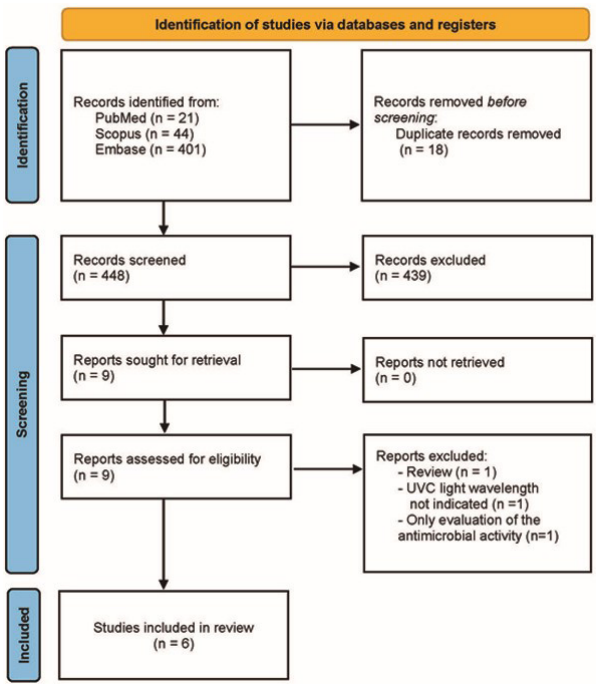
Flowchart of the review to identify included studies.

The electronic search identified 466 articles from three databases (21 in PubMed, 44 in Scopus, and 401 in Embase). After duplicates had been removed, 448 articles were screened. After reading the title and abstracts, 439 additional articles were excluded. All articles were retrieved; thus 9 articles were full text screened for eligibility. Finally, 6 articles were selected and included for final evaluation. Figure 1 shows the flowchart of this scoping review and the detailed information of included studies is presented in  Table 1.

The dimensional stability of dental impression materials was evaluated by measuring dental casts made from an impression (24-26,28) or measuring distances on discs made from stainless steel dies (27,29). For the evaluation, most studies considered the term “dimensional stability” (24-28); only one study used the term “dimensional accuracy” (29). The included studies reported the use of polyether, addition and condensation silicones, alginate, and zinc oxide eugenol (24-29). According to the sample size, 10 dental casts (25,26,28), 20 dental casts (24) or 10 discs (27,29) were used per group. Regarding the disinfection duration in the UVC light chamber, alginate was exposed to 3 (25) and 10 minutes (29); addition silicone to 3, 10, 20, and 40 minutes (24-27,29); condensation silicone to 40 minutes (27); polyether to 10 (29) and 20 minutes (28), and zinc oxide eugenol to 10 minutes (29). All the studies used a wavelength of 254 nm.

Most measuring devices considered in the studies were traveling microscopes (24-26); one study adopted a digital caliper (27), another a coordinate measuring machine capable of recording on the X- and Y-axes (28), and another a video measuring machine with a geometric measuring software (29). Regarding the measurements, three studies considered anteroposterior and cross-arch distances (24,26,28), one study reported inter abutment distance, cross-arch distance, and occluso-gingival length (25), and two studies included the distances between lines on the surface of the ruled blocks and specimens (28,29). The included studies recommended using UVC light for the disinfection of polyether, and addition and condensation silicones without significative dimensional changes.

## Discussion

Dental impressions are negative imprints of hard and soft oral tissues and are considered the first milestone in the sequence of procedures performed to fabricate dental casts (4). In the oral cavity, these materials come in contact with saliva, blood, debris, and dental plaque which contain some virus and pathogenic micro-organisms (6). Thus, disinfection is mandatory to minimize the risk of disease transmission in the patients, dental staff, and laboratory technicians, and different methods have been proposed for that objective. One of these methods is UVC light, a physical method with substantial evidence in the literature (10,15,17,18). However, considering the main requirements of the disinfection methods, the dental impression materials should remain dimensionally stable after the disinfection process. This scoping review comprehensively assessed the information about the effect of UVC light on the dimensional stability of dental impression materials.

Chidambaranathan and Balasubramanium (10) performed a review of the techniques available in the dental realm for dental impression disinfection and listed the use of iodophor, glutaraldehyde, sodium hypochlorite, quaternary ammonium chloride salt, alcohols (isopropyl and ethyl alcohol), chlorhexidine, ozone water, direct current glow discharge, steam autoclave, ethylene oxide gas autoclave, argon radio frequency glow discharge, microwave irradiation, and UV light. In addition to the information presented previously, in a recent meta-analysis, Hardan *et al*. (30) added the use of hydrogen peroxide, povidone iodine, single or dual quaternary ammonium compound, silver nanoparticles, sodium dichloroisocyanurate, sodium hypochlorite with sodium chloride, and electrolyzed oxidizing water as existing disinfection procedures used against micro-organisms colonization on dental impression materials. According to this scoping review, three of the six included studies compared the effect of UVC light against glutaraldehyde 2% and sodium hypochlorite (1% or 5.25%) (25,26,28), one study compared the UVC light against quaternary ammonium salts, phenoxyethanol, alcohol, and ozone (27). Meanwhile, another study compared the effect of UVC light against peracetic acid 0.2%, natural polymer of glucosamine and ozonated water (29). It is interesting to notice that most of the disinfection methods listed in this scoping review have been recommended to be employed to disinfect alginate, polyvinyl siloxane, and polyether (30).

Considering the protocols, Godbole *et al*. (24) and Nimonkar *et al*. (26) performed the same technique using a modified articulator with free condylar housing, permitting only the opening and closing movements. Furthermore, the contact of the incisal guide pin with the guide Table enabled to maintain the distances between the custom tray and the master mold that, which helped to standardize the thickness of the impression material for all the specimens. Samra and Bhide (25) considered a mounting jig with two fiberglass plates that included guide posts and metal stops to ensure that the amount of impression material was uniform for every impression made. Joshy *et al*. (28) used three stoppers (two in the back and one in the front) placed on the land area of the mandibular master model to ensure proper alignment of the impression trays for the open-tray dental implant impression technique. Following another technique, Wezgowiec *et al*. (27) and Sabharwal *et al*. (29) performed protocols using stainless steel dies based on dental stone. The impression technique followed by Samra and Bhide (25) for the addition silicone evaluation was a one-step putty wash technique; that is, putty material mixed and loaded into the tray while light body addition silicone was injected into the metal die through automatic dispenser. Conversely, Godbole *et al*. (24) and Nimonkar *et al*. (26) used a two-step putty wash considering a polyethylene spacer.

Regarding the disinfection duration in the UVC light chamber, the included studies contemplated diverse times. One study used 3 minutes for alginate and addition silicone (25), a different study considered 10 minutes for addition silicone (24), another study considered 10 minutes for zinc oxide eugenol, alginate, and polyether (29), two studies examined 20 minutes for addition silicone and polyether (26,28), and one study used 40 minutes for addition and condensation silicones (27). According to the literature, there are investigations that evaluated the UVC light disinfection potential considering 15, 30, 60, 90, 120, and 180 seconds for addition silicone (31), 3 minutes for alginate and addition silicone (32), 20 minutes for alginate (33), 40 minutes for addition and condensation silicones (18), and 3, 6, 10, and 15 minutes for alginate, addition silicone, and polyether (17). This latter investigation performed by Aeran *et al*. (17) concluded that for alginate and addition silicone, disinfection was achieved considering 10 minutes of exposure; meanwhile for polyether, 3 minutes were sufficient to produce complete disinfection. Bearing in mind the diversity of the UVC light disinfection time reported, further studies need to be done in order to standardize the duration of the exposition considering the dental impression material used. In addition, UVC light irradiance and distance play essential roles in the efficacy of the disinfection. Godbole *et al*. (24) and Nimonkar *et al*. (26) highlighted the importance of proper application and the short effective range of UVC light irradiance, typically 10 cm (distance between impression tray and UVC light source).

Considering the master mold and the measurements performed, two studies reported the use of a brass mold simulating a maxillary arch with five tapered abutments with pointed tips (24,26), one study considered a maxillary arch stainless steel die designed by CAD/CAM technology (25), other study reported the use of a mandibular arch heat-cured acrylic cast (28), and two studies used stainless steel dies based on dental stones (27,29). The included studies compared linear measurements using a traveling microscope (24-26), a digital caliper (27), a machine capable of recording on the X- and Y-axes (28), or a video measuring machine with a geometric measuring software (29). The studies that evaluated dental casts used measurements of anteroposterior and cross-arch distances (24-26,28); only Samra and Bhide (25) included occluso-gingival length. The most recently published study (29) used a measuring software compared to others that used more conventional measuring methods. It is essential to point out that one study evaluated two alginates from different manufacturers and found significant dimensional changes in one of them (25). Furthermore, another study reported that the dimensional stability of zinc oxide eugenol and alginate was significantly affected after exposure to UVC light (29). Polyether and addition and condensation silicones showed the best results without compromising the dimensional stability.

Many protocols considered horizontal linear measurements. Researchers should include three-dimensional measurements in future investigations utilizing customized software. In addition, the disinfection duration in the UVC light chamber without compromise other dental properties such as hardness, wettability, tensile strength, surface roughness, and detail reproduction should be further studied in future because these are important characteristics that influence the clinical performance of dental materials.

## Conclusions

The following conclusions could be drawn based on the findings of this scoping review:

- Most measurements of dental impression materials showed contraction and expansion or only contraction among the reference points evaluated, but clinically insignificant.

- UVC light showed no significant dimensional changes in polyether, and addition and condensation silicones.

- UVC light can be a recommended method to disinfect alginates used for procedures that do not require high precision. Zinc oxide eugenol material showed the most significant dimensional changes.

## Figures and Tables

**Table 1:** Description of the studies included in the review.

Author /year	Impression material	Sample size (dental casts or discs)	Disinfection duration in the UVC light chamber	Groups evaluated	Measurement equipment	Measurements	Results
Godbole et al. (24) / 2014	Addition silicone	40 dental casts (20 per group)	10 minutes at 254 nm	Control and UVC	Traveling microscope of 0.001mm accuracy	Anteroposterior and cross-arch distances	UVC light showed no significant dimensional changes on impressions
Samra and Bhide (25) / 2018	Alginate and addition silicone	40 dental casts (10 per group)	3 minutes at 254 nm	Control, glutaraldehyde 2%, sodium hypochlorite 5.25% and UVC	Traveling microscope of 10 um of accuracy	Interabutment distance, cross-arch distance, occluso-gingival length	UVC chamber can be a recommended method for disinfecting impressions without compromising their dimensional stability
Nimonkar et al. (26) / 2019	Addition silicone	40 dental casts (10 per group)	20 minutes at 254 nm	Control, glutaraldehyde 2%, sodium hypochlorite 1% and UVC	Traveling microscope of 0.001mm accuracy	Anteroposterior and cross-arch distances	UVC light showed no significant dimensional changes on impressions
Wezgowiec et al. (27) / 2022	Addition and condensation silicones	50 discs (10 per group)	40 minutes at 254 nm	Control, immersion solution (quaternary ammonium salts, phenoxyethanol), spray (alcohols), ozone and UVC	Digital caliper	Distance between lines d1 and d2 along line c	UVC did not significantly influence the dimensional stability of studied dental silicones
Joshi et al. (28) / 2024	Polyether	40 implant dental casts (10 per group)	20 minutes at 254 nm	Control, glutaraldehyde 2%, sodium hypochlorite 1% and UVC	Coordinate measuring machine capable of recording on the X- and Y-axes	Anteroposterior and cross-arch distances	UVC light maintained dimensional stability on impressions
Sabharwal et al. (29) / 2024	Zinc oxide eugenol, alginate, polyether, addition silicone	40 discs (10 per group)	10 minutes at 254 nm	Peracetic acid 0.2%, natural polymer of glucosamine, ozonated water and UVC	Video measuring machine with a geometric measuring software	Distance between lines A and B along line X	Polyether and addition silicone can be disinfected with UVC without causing a significant change in dimensional accuracy

## Data Availability

The datasets used and/or analyzed during the current study are available from the corresponding author.

## References

[1] Ud Din S, Sajid M, Saeed A, Chaudhary FA, Alam MK, Sarfraz J (2022). Dimensional changes of commercial and novel polyvinyl siloxane impression materials following sodium hypochlorite disinfection. PeerJ.

[2] Qiu Y, Xu J, Xu Y, Shi Z, Wang Y, Zhang L (2023). Disinfection efficacy of sodium hypochlorite and glutaraldehyde and their effects on the dimensional stability and surface properties of dental impressions: a systematic review. PeerJ.

[3] Rubel BS (2007). Impression materials: a comparative review of impression materials most commonly used in restorative dentistry. Dent Clin North Am.

[4] Punj A, Bompolaki D, Garaicoa J (2017). Dental impression materials and techniques. Dent Clin North Am.

[5] Jayaraman S, Singh BP, Ramanathan B, Pazhaniappan Pillai M, MacDonald L, Kirubakaran R (2018). Final-impression techniques and materials for making complete and removable partial dentures. Cochrane Database Syst Rev.

[6] Azevedo MJ, Correia I, Portela A, Sampaio-Maia B (2019). A simple and effective method for addition silicone impression disinfection. J Adv Prosthodont.

[7] Flanagan DA, Palenik CJ, Setcos JC, Miller CH (1998). Antimicrobial activities of dental impression materials. Dent Mat.

[8] Badrian H, Ghasemi E, Khalighinejad N, Hosseini N (2012). The effect of three different disinfection materials on alginate impression by spray method. ISRN Dent.

[9] Ganavadiya R, Chandra Shekar BR, Saxena V, Tomar P, Gupta R, Khandelwal G (2014). Disinfecting efficacy of three chemical disinfectants on contaminated diagnostic instruments: A randomized trial. J Basic Clin Pharm.

[10] Chidambaranathan AS, Balasubramanium M (2019). Comprehensive review and comparison of the disinfection techniques currently available in the literature. J Prosthodont.

[11] Al Mortadi N, Al-Khatib A, Alzoubi KH, Khabour OF (2019). Disinfection of dental impressions: knowledge and practice among dental technicians. Clin Cosmetic and Investig Dent.

[12] Kotsiomiti E, Tzialla A, Hatjivasiliou K (2008). Accuracy and stability of impression materials subjected to chemical disinfection-a literature review. J Oral Rehabil.

[13] Naumovski B, Kapushevska B (2017). Dimensional stability and acuracy of silicone-based impression materials using different impression techniques-A literature review. Pril (Makedon Aka Nauk Umet Odd Med Nauki).

[14] Saini RS, Alshadidi AAF, Hassan SAB, Aldosari LIN, Mosaddad SA, Heboyan A (2024). Properties of a novel composite elastomeric polymer vinyl polyether siloxane in comparison to its parent materials: A systemic review and meta-analysis. BMC Oral Health.

[15] AlZain S (2019). Effect of chemical, microwave irradiation, steam autoclave, ultraviolet light radiation, ozone and electrolyzed oxidizing water disinfection on properties of impression materials: A systematic review and meta-analysis study. Saudi Dent J.

[16] Bhardwaj SK, Singh H, Deep A, Khatri M, Bhaumik J, Kim KH (2021). UVC-based photoinactivation as an efficient tool to control the transmission of coronaviruses. Sci Total Environ.

[17] Aeran H, Sharma S, Kumar V, Gupta N (2015). Use of clinical UV chamber to disinfect dental impressions: A comparative study. J Clin Diagn Res.

[18] Wezgowiec J, Wieczynska A, Wieckiewicz M, Czarny A, Malysa A, Seweryn P (2022). Evaluation of antimicrobial efficacy of UVC radiation, gaseous ozone, and liquid chemicals used for disinfection of silicone dental impression materials. Materials.

[19] Montalli VAM, Freitas PR, Torres MF, Torres Junior OF, Vilhena DHM, Junqueira JLC (2021). Biosafety devices to control the spread of potentially contaminated dispersion particles. New associated strategies for health environments. PloS One.

[20] Agrawal SK, Dahal S, Bhumika TV, Nair NS (2019). Evaluating sanitization of toothbrushes using various decontamination methods: A meta-analysis. J Nepal Health Res Counc.

[21] Binns R, Li W, Wu CD, Campbell S, Knoernschild K, Yang B (2020). Effect of ultraviolet radiation on candida albicans biofilm on poly (methylmethacrylate) resin. J Prosthodont.

[22] Jalaluddin M, Ramanna PK, Swain M, Sonkesriya S, Rana P, Kumari D (2024). Evaluation of fibrin clot interaction with dental implant after different surface treatments: An in vitro study. J Contemp Dent Pract.

[23] Tricco AC, Lillie E, Zarin W, O'Brien KK, Colquhoun H, Levac D (2018). PRISMA extension for scoping reviews (PRISMA-ScR): Checklist and explanation. Ann Intern Med.

[24] Godbole SR,  Dahane TM, Patidar NA, Nimonkar SV (2014). Evaluation of the effect of ultraviolet disinfection on dimensional stability of the polyvinyl silioxane impressions. An in-vitro study. J Clin Diagn Res.

[25] Samra RK, Bhide SV (2018). Comparative evaluation of dimensional stability of impression materials from developing countries and developed countries after disinfection with different immersion disinfectant systems and ultraviolet chamber. Saudi Dent J.

[26] Nimonkar SV, Belkhode VM, Godbole SR, Nimonkar PV, Dahane T, Sathe S (2019). Comparative evaluation of the effect of chemical disinfectants and ultraviolet disinfection on dimensional stability of the polyvinyl siloxane impressions. J Int Soc Prev Community Dent.

[27] Wezgowiec J, Paradowska-Stolarz A, Malysa A, Orzeszek S, Seweryn P, Wieckiewicz M (2022). Effects of various disinfection methods on the material properties of silicone dental impressions of different types and viscosities. Int J Mol Sci.

[28] Joshi S, Madhav VNV, Saini RS, Gurumurthy V, Alshadidi AAF, Aldosari LIN (2024). Evaluation of the effect of chemical disinfection and ultraviolet disinfection on the dimensional stability of polyether impression material: an in-vitro study. BMC Oral Health.

[29] Sabharwal N, Arora A, Upadhyaya V, Sehgal MM, Nayak K, Katyal S (2024). Impression disinfection and its effect on dimensional accuracy and surface detail in the times of COVID-19: An in vitro study. Cureus.

[30] Hardan L, Bourgi R, Cuevas-Suárez CE, Lukomska-Szymanska M, Cornejo-Ríos E, Tosco V (2022). Disinfection procedures and their effect on the microorganism colonization of dental impression materials: A systematic review and meta-analysis of in vitro studies. Bioengineering.

[31] Anand V (2013). A comparative evaluation of disinfection effect of exposures to ultra-violet light and direct current glow discharge on Candida Albicans colonies coated over elastomeric impression material: An in vitro study. J Pharm Bioallied Sci.

[32] Samra RK, Bhide SV (2010). Efficacy of different disinfectant systems on alginate and addition silicone impression materials of Indian and international origin: a comparative evaluation. J Indian Prosthodont Soc.

[33] Kotwal M, Singh VP, Mushtaq H, Ahmed R, Rai G, Kumar A (2021). Disinfection of impression materials with glutaraldehyde, ultraviolet radiation, and autoclave: A comparative study. J Pharm Bioallied Sci.

